# Cell Membrane-Coated Biomimetic Nanocarriers for Plaque-Targeted Atherosclerosis Therapy: Molecular Mechanisms, Inflammatory Microenvironments, and Translational Challenges

**DOI:** 10.3390/ijms27177718

**Published:** 2026-08-28

**Authors:** Ruiyu Zhang, Xujing Yuan, Jinpeng Sun, Yumiao Wei

**Affiliations:** 1 Department of Cardiology, Union Hospital, Tongji Medical College, Huazhong University of Science and Technology, Wuhan 430022, China; 2 Hubei Key Laboratory of Biological Targeted Therapy, Union Hospital, Tongji Medical College, Huazhong University of Science and Technology, Wuhan 430022, China; 3 Hubei Provincial Engineering Research Center of Immunological Diagnosis and Therapy for Cardiovascular Diseases, Union Hospital, Tongji Medical College, Huazhong University of Science and Technology, Wuhan 430022, China; 4 Key Laboratory of Biological Targeted Therapy (Huazhong University of Science and Technology), Ministry of Education, Wuhan 430022, China

**Keywords:** atherosclerosis, cell membrane-coated nanoparticles, biomimetic nanocarriers, plaque targeting, targeted therapy, inflammatory microenvironment, drug delivery

## Abstract

Atherosclerosis (AS) is a lipid-driven chronic inflammatory disease and the principal pathological basis of coronary artery disease, ischemic stroke, and peripheral artery disease. Despite advances in lipid-lowering, antiplatelet, anti-inflammatory, and revascularization strategies, current treatments do not selectively modulate the heterogeneous cellular and inflammatory microenvironments within individual plaques. Cell membrane-coated nanoparticles (CMNs) have therefore emerged as biomimetic delivery platforms that combine the functional versatility of synthetic nanocores with selected biological properties of donor-cell membranes. This review summarizes the fabrication of CMNs and critically compares platelet-, macrophage-, neutrophil-, monocyte-, erythrocyte-, and hybrid membrane-coated nanoplatforms, together with emerging T-cell-targeted nanotherapeutic strategies. Particular attention is given to membrane-source selection, receptor–ligand interactions, immune evasion, lesion-directed accumulation, stimulus-responsive cargo release, and the modulation of inflammation, oxidative stress, lipid metabolism, and defective efferocytosis. We further discuss major translational barriers, including donor- and activation-state-dependent membrane heterogeneity, incomplete or unstable coating, manufacturing reproducibility, scalability, thrombogenicity, immunogenicity, and the limited predictive value of short-term small-animal models. A pathology-matched and minimum-sufficient-complexity framework is proposed to guide the rational development of safer, more reproducible, and clinically relevant biomimetic nanotherapies for AS.

## 1. Introduction

Atherosclerosis (AS) is a lipid-driven chronic inflammatory disease of large and medium-sized arteries and constitutes the principal pathological basis of coronary artery disease, ischemic stroke, and peripheral artery disease, collectively imposing a substantial global health burden [1]. Its development involves the subendothelial retention and modification of apolipoprotein B-containing lipoproteins, endothelial activation, immune-cell recruitment, macrophage foam-cell formation, vascular smooth muscle-cell phenotypic modulation, defective efferocytosis, and progressive remodeling of the extracellular matrix [2,3]. These interconnected processes generate heterogeneous plaque microenvironments that differ substantially in cellular composition, inflammatory activity, lipid burden, necrotic-core formation, fibrous-cap integrity, and thrombotic risk.

Current prevention and treatment strategies primarily include comprehensive risk-factor control, lipid-lowering therapy, antiplatelet treatment in appropriately selected patients, and revascularization when clinically indicated [4,5]. Although these strategies have substantially reduced cardiovascular risk, conventional pharmacotherapies are generally administered systemically and do not selectively modulate the heterogeneous cellular and inflammatory microenvironments within individual plaques. Residual inflammatory risk, defective efferocytosis, persistent lipid accumulation, and incomplete stabilization of high-risk plaques therefore remain important therapeutic challenges [6,7,8]. Anticoagulant and thrombolytic agents are reserved for specific acute thrombotic conditions rather than routine modification of underlying atherosclerotic lesions [8,9].

These unmet therapeutic needs have stimulated the development of delivery strategies capable of improving lesion-selective drug exposure and locally modulating the atherosclerotic plaque microenvironment. Nanocarrier-based delivery systems have consequently been investigated as potential platforms for improving the spatial and temporal control of anti-atherosclerotic therapy [10,11]. Compared with administration of the corresponding free drug, appropriately designed nanocarriers may improve cargo stability and pharmacokinetic behavior, increase local drug exposure, and reduce off-target exposure [12,13,14]. Depending on their composition and cargo, nanocarriers can improve the apparent solubilization of poorly water-soluble compounds, protect labile therapeutic agents from premature degradation, and provide controlled or sustained release. Nanoparticles can also be functionalized with plaque-recognition ligands or biomimetic surfaces to enhance interactions with activated endothelium, inflammatory macrophages, foam cells, or thrombus-associated components. Separately, stimulus-responsive nanocarriers can exploit pathological cues such as elevated reactive oxygen species, acidic pH, or disease-associated enzymes to promote site-specific cargo release [15]. Together, these features provide opportunities for improved spatial and temporal control of therapeutic interventions within atherosclerotic plaques. Despite these advantages, many synthetic nanoparticles exhibit limited biological specificity and are readily opsonized and cleared by the mononuclear phagocyte system, resulting in substantial sequestration in the liver and spleen and insufficient delivery to atherosclerotic lesions [10,16]. Polyethylene glycol (PEG) modification is the most widely used strategy for improving nanoparticle biocompatibility and prolonging circulation [17]. However, pre-existing or treatment-induced anti-PEG antibodies may alter nanoparticle pharmacokinetics, promote accelerated blood clearance after repeated administration, and contribute to hypersensitivity reactions in susceptible individuals [18,19].

Cell membrane coating has therefore emerged as a promising alternative surface-passivation strategy [20,21]. In this approach, synthetic nanoparticles are cloaked with natural cell membranes, allowing them to mimic endogenous cells. This biomimetic camouflage helps the particles evade immune clearance and thereby prolongs circulation, while the retained functional membrane proteins confer additional biological properties [22]. For example, leukocyte-associated integrins and selectin ligands may interact with vascular cell adhesion molecule-1 (VCAM-1), intercellular adhesion molecule-1 (ICAM-1), and selectins upregulated on activated endothelium, whereas platelet-associated receptors can recognize von Willebrand factor, exposed collagen, fibrinogen, and fibrin within injured or thrombotic vascular regions [23]. In addition, nanoparticle-associated cluster of differentiation 47 (CD47) can bind signal regulatory protein alpha (SIRPα) on macrophages and suppress phagocytosis, thereby extending blood residence time [24]. Owing to these properties, cell membrane-coated nanoparticles (CMNs) have increasingly been investigated for biologically guided treatment of cardiovascular diseases, particularly atherosclerosis [15,20].

The importance of cell membrane coating lies in the fact that it is not merely a surface modification; rather, it endows nanomaterials with a biological identity and transforms them from passive carriers into actively functionalized platforms. Specifically, (1) preservation of intact membrane proteins enables nanoparticles to mimic the homing behavior of donor cells [25,26], facilitating directed transendothelial migration and retention within plaques; (2) the coordinated action of multiple functional proteins can simultaneously confer immune evasion, targeted adhesion, and microenvironmental regulation, thereby overcoming the limited efficiency of single-ligand modification [27,28]; (3) membrane fluidity and plasticity provide a structural basis for integrating functional modules derived from multiple cell types, giving rise to next-generation designs such as hybrid membranes. Collectively, these advantages make cell membrane coating a key strategy for overcoming current bottlenecks in nanomedicine-based treatment of AS [15,20].

Existing reviews have largely summarized cell membrane-coated nanoplatforms according to membrane source, carrier composition, or therapeutic modality [10,15,20], whereas comparative analysis of how different membrane types correspond to specific plaque microenvironments remains limited. This review therefore adopts a pathology-matched framework that links plaque-associated cellular and molecular niches with membrane-source selection, receptor–ligand interactions, therapeutic functions, evidence maturity, and translational barriers. Particular emphasis is placed on studies published during the past five years, while seminal earlier reports are included where necessary to establish the development of the field. By comparing the compatibility of different membrane sources with specific pathological processes and their corresponding functional advantages and limitations, we aim to inform the rational design and clinical translation of future biomimetic nanodelivery systems for atherosclerosis.

## 2. Literature Search Strategy

A structured literature search was conducted using PubMed, Web of Science, and Google Scholar. The primary search focused on peer-reviewed studies published between 2021 and 2026, while selected earlier seminal studies were additionally included when necessary to establish key technological developments or fundamental mechanistic concepts.

The search strategy was organized around five major concepts: “atherosclerosis”, “nanoparticle”, “biomimetic”, “cell membrane”, and “targeting”. These terms were used in various combinations to capture a broad range of relevant studies. Additional source-specific terms, including “platelet membrane”, “macrophage membrane”, “neutrophil membrane”, “monocyte membrane”, “erythrocyte membrane”, and “hybrid membrane”, were combined with “atherosclerosis” and nanoparticle-related terms. Targeted searches were also conducted for topics such as membrane coating, membrane engineering, coating integrity, and translational quality control.

Peer-reviewed English-language original studies directly addressing cell membrane-based biomimetic nanoplatforms for atherosclerosis were prioritized. Mechanistic or methodological studies from other disease models were also considered when directly relevant to membrane fabrication, biological functionality, or quality control. High-quality reviews and guidelines were used primarily for background information. Duplicate records, conference abstracts, editorials, non-peer-reviewed reports, and studies lacking substantive relevance were excluded. Titles and abstracts were screened initially, followed by full-text assessment of potentially relevant publications.

## 3. Pathological Mechanisms of Atherosclerosis and the Rationale for Targeting with Biomimetic Nanomaterials

Atherosclerosis is a chronic inflammatory disease of large arteries and is a major pathological basis of cardiovascular disease and stroke [29]. It is characterized by the accumulation of lipid-rich plaques within the arterial wall. Progressive plaque growth can narrow the arterial lumen, whereas disruption of high-risk plaques can precipitate thrombosis and acute ischemic events [30]. Atherosclerotic cardiovascular disease remains a major contributor to global morbidity and mortality [31]. Its prevalence increases markedly with age, although subclinical coronary atherosclerosis is already common in asymptomatic middle-aged adults [32]. Its incidence has increasingly shifted toward younger populations [33]. Major risk factors include hypertension, hyperlipidemia, smoking, diabetes mellitus, obesity, and genetic susceptibility [34].

Key pathogenic events in AS include endothelial dysfunction, leukocyte adhesion, macrophage foam-cell formation, and phenotypic switching of vascular smooth muscle cells (VSMCs) [35]. Aberrant endothelial activation compromises barrier integrity and weakens intercellular junctions, thereby facilitating the entry of low-density lipoprotein (LDL) into the subendothelial space and the formation of fatty-streak deposits [36]. Concomitantly, activated endothelial cells upregulate adhesion molecules and present chemokines that coordinate leukocyte homing, adhesion, and transendothelial migration, particularly the recruitment of monocytes into the arterial wall [37,38]. Monocytes recruited to the arterial intima differentiate into inflammatory macrophages. Through scavenger receptors, these cells internalize lipoprotein particles such as oxidized low-density lipoprotein (oxLDL), accumulate cholesterol, and transform into foam cells. T lymphocytes and other immune cells also migrate into lesions, where they modulate innate immune and endothelial-cell functions and amplify plaque inflammation. VSMCs likewise play a pivotal role in plaque development. Activated VSMCs migrate from the media into the intima and undergo a phenotypic transition from a contractile to a synthetic state [39]. They can subsequently adopt multiple cell-like phenotypes, including foam-cell-like and fibromyocyte-like states, and contribute to neointimal formation, fibrous-plaque development, arterial calcification, and ultimately the formation of the fibrous cap overlying the plaque [40]. As plaques progress, VSMCs and macrophage-derived foam cells may undergo apoptosis. The accumulation of uncleared cellular debris gives rise to a lipid-rich necrotic core [41,42], and inflammatory mediators released from this region further intensify responses in neighboring cells. Vulnerable, or “unstable,” plaques typically contain a large lipid core covered by a thin fibrous cap (<65 μm) [43]. Such plaques are highly prone to rupture and may trigger acute thrombotic events [44].

**Figure 1:**
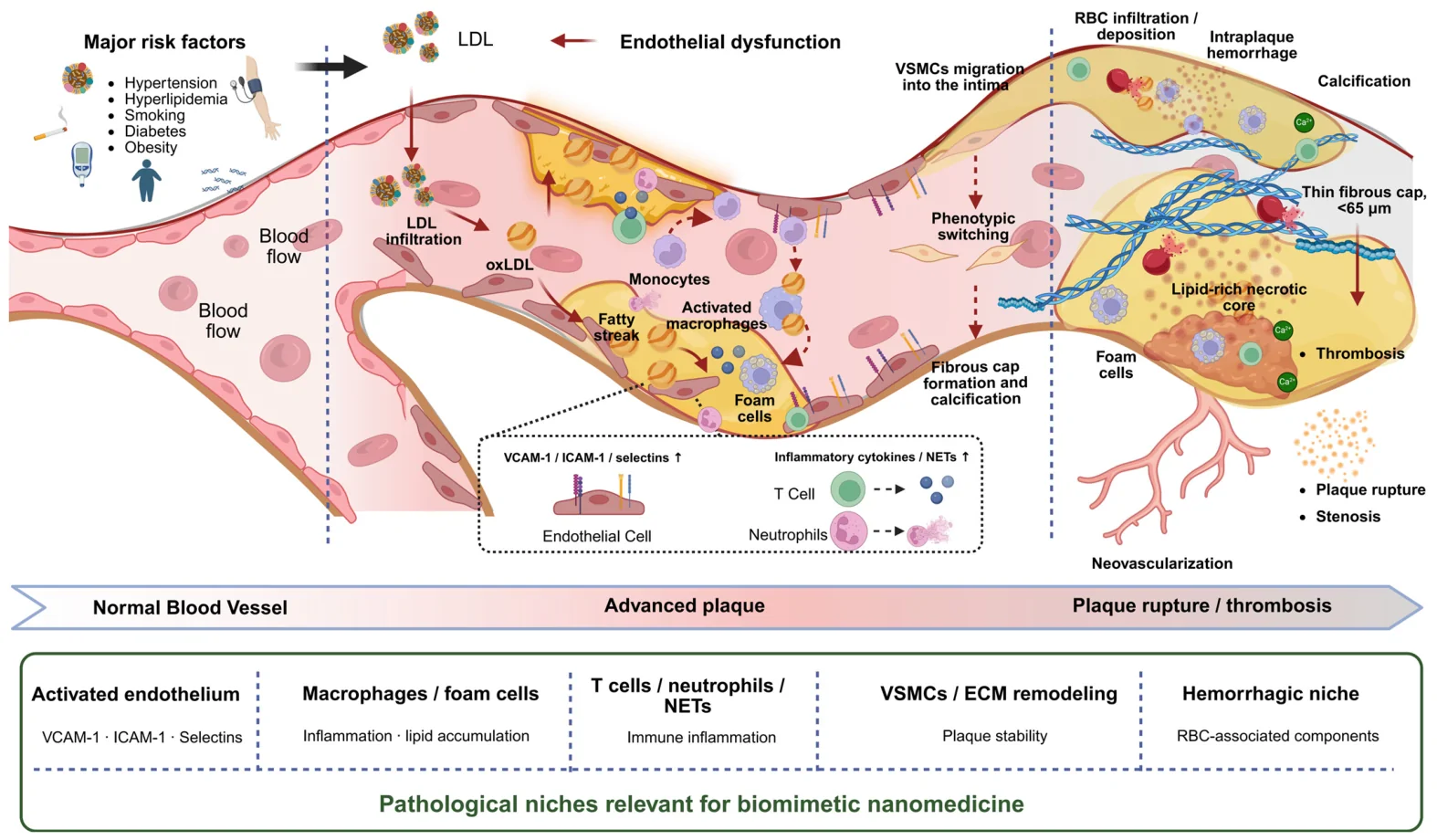
Pathological progression of atherosclerosis and targetable pathological niches relevant to biomimetic nanomedicine. Cardiovascular risk factors initiate endothelial dysfunction, lipid accumulation, immune-cell recruitment, and inflammatory amplification, leading to plaque progression, instability, and thrombosis. The major pathological niches associated with biomimetic nanomedicine include activated endothelium, macrophage/foam-cell-rich regions, inflammatory immune niches, vascular smooth muscle cell/extracellular matrix remodeling regions, and hemorrhagic microenvironments. Abbreviations: LDL, low-density lipoprotein; oxLDL, oxidized low-density lipoprotein; VCAM-1, vascular cell adhesion molecule-1; ICAM-1, intercellular adhesion molecule-1; NETs, neutrophil extracellular traps; VSMCs, vascular smooth muscle cells; ECM, extracellular matrix; RBC, red blood cell.

From the perspective of nanodelivery design, an atherosclerotic plaque is not simply a focal lipid deposit but a complex pathological microenvironment comprising activated endothelium, inflammatory immune cells, foam cells, VSMCs, extracellular matrix, a necrotic core, neovessels, calcified regions, and intraplaque hemorrhage. Each component provides a distinct biological niche that can be exploited for pathology-matched localization and therapeutic intervention by cell membrane-based biomimetic platforms [15]. Adhesion molecules upregulated on activated endothelial cells, including VCAM-1, ICAM-1, P-selectin, and E-selectin, provide a molecular basis for inflammation-associated adhesion and transendothelial migration mediated by platelet, neutrophil, and monocyte membranes. Monocytes/macrophages and foam cells enriched within plaques constitute the core sites of inflammatory amplification and dysregulated lipid metabolism, supporting the use of macrophage membrane-coated nanomaterials to achieve inflammatory homing, regulate cholesterol efflux, and enhance efferocytosis. T cells and neutrophils contribute to inflammatory amplification, neutrophil extracellular trap (NET) formation, and fibrous-cap destabilization, thereby providing potential targets for immunomodulatory membrane-coated nanoplatforms. VSMC phenotypic switching, abnormal collagen metabolism, and matrix metalloproteinase-mediated extracellular-matrix degradation determine fibrous-cap stability and are therefore central to plaque-stabilizing therapy. Finally, rupture of intraplaque neovessels leads to erythrocyte deposition, release of cell-free hemoglobin, and increased cholesterol burden, providing a rationale for the use of erythrocyte membranes and erythrocyte–platelet hybrid membranes to prolong circulation and enhance lesion accumulation. Accordingly, CMNs should not be viewed merely as surface-camouflage systems, but as biomimetic delivery platforms rationally matched to the cellular composition, molecular adhesion network, and inflammatory microenvironment of atherosclerotic plaques (Figure 1).

## 4. Cell Membrane-Coated Nanoparticles

Cell membrane-coated nanoparticles represent a highly biomimetic technology that combines the isolation of natural cell membranes with the fabrication of synthetic nanoparticle cores [20,45,46]. In a typical CMN architecture, membrane vesicles derived from erythrocytes, macrophages, other leukocytes, cancer cells, or related cellular sources form a biomimetic outer layer around nanoscale cores composed of polymeric, lipid-based, metallic, or other inorganic materials [47]. Studies have also shown that living-cell membranes can coat nanoparticles through relatively simple, non-destructive centrifugation-based approaches that mimic the endocytic and exocytic processes involved in extracellular-vesicle release [48]. This core–shell configuration preserves the biological properties of the cell membrane while retaining the functional versatility of synthetic nanoparticles, offering distinctive advantages for drug delivery, including favorable biocompatibility, prolonged circulation, immune evasion, and homotypic targeting [49]. Although substantial translational challenges remain [50], CMNs have broad potential in medicine and pharmaceutical development [51].

### 4.1. Preparation Methods and Workflow

**Figure 2:**
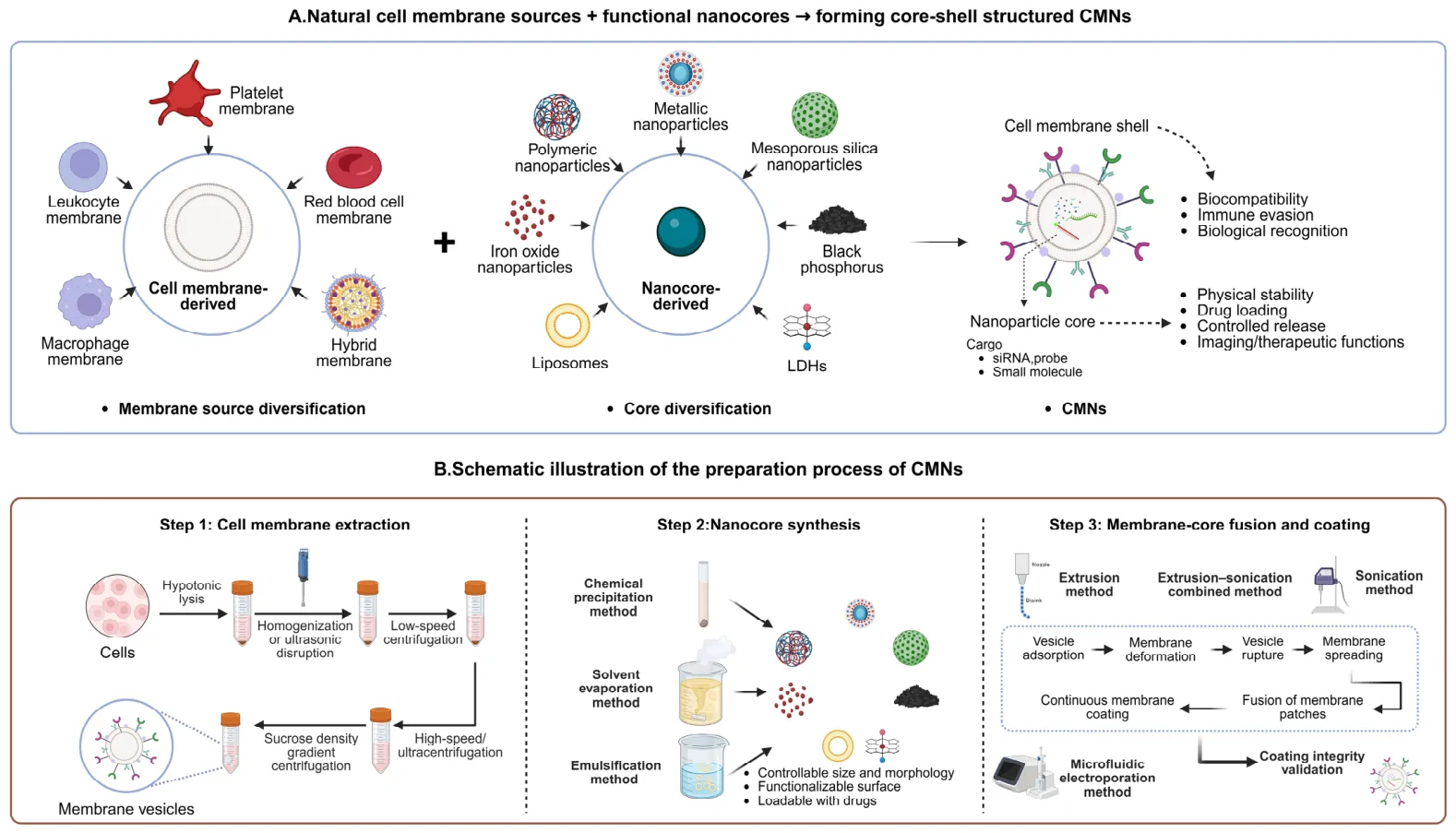
Schematic illustration of the structural components and fabrication strategies of cell membrane-coated nanoparticles (CMNs). Natural cell membranes from different sources can be combined with diverse nanocores to generate biomimetic core–shell nanoparticles. The fabrication process mainly involves membrane isolation, nanocore preparation, and membrane–core fusion/coating through approaches such as extrusion, sonication, electroporation, and microfluidic-assisted methods. Abbreviations: CMNs, cell membrane-coated nanoparticles; LDHs, layered double hydroxides.

The preparation of CMNs generally involves three principal steps: cell membrane extraction, synthesis of the nanoparticle core, and integration of the membrane with the core [20,48] (Figure 2).

#### 4.1.1. Cell Membrane Extraction

A standard membrane-isolation workflow typically comprises sequential hypotonic lysis, homogenization or sonication, low-speed centrifugation to remove nuclei and large debris, high-speed or ultracentrifugation to pellet crude membranes, and final purification by resuspension followed by sucrose-density-gradient centrifugation to collect the purified membrane-vesicle fraction [46,48]. In practice, cells are first physically disrupted to release intracellular contents and are then purified through a series of differential-centrifugation steps. Low-speed centrifugation removes cellular debris, whereas high-speed density-gradient centrifugation isolates intact membrane vesicles. These vesicles preserve the native architecture and surface antigens of the donor-cell membrane, including glycoprotein receptors and ligands, and can therefore mimic natural cell–cell interactions [52]. Centrifugation is simple, efficient, and capable of yielding membrane vesicles without extensive chemical damage; however, operating conditions must be carefully optimized to preserve membrane integrity while maintaining high purity and yield. Nitrogen cavitation performed in isotonic buffer, combined with sucrose-gradient separation [53], can improve the quality and purity of membrane preparations used for CMN fabrication [54,55]. Magnetic-bead-assisted enrichment has also emerged as a method for selectively isolating highly purified membrane fractions.

#### 4.1.2. Nanoparticle-Core Synthesis

Nanoparticle cores are generally prepared using established chemical methods that allow precise control over particle size and morphology. Common approaches include chemical precipitation, solvent evaporation, and emulsification [56]. Chemical precipitation is frequently used for metallic nanoparticles such as gold or silver, in which reducing agents induce the nucleation and precipitation of metal ions to generate particles with relatively uniform dimensions [57]. Solvent evaporation is commonly applied to polymeric nanoparticles such as poly(lactic-co-glycolic acid) (PLGA): the polymer is first dissolved in an organic solvent, emulsified into microdroplets, and then solidified as the solvent evaporates [58]. Emulsification methods are used to prepare emulsion-based nanocarriers by mixing aqueous and oil phases under high shear and stabilizing the resulting nanoparticles with surfactants [59]. These methods are intended to achieve monodispersity, controllable size, and surface functionalization, often through the addition of stabilizers or surface ligands that enhance biocompatibility.

A wide range of nanocarriers has been developed, including liposomes, gold nanoparticles, mesoporous silica nanoparticles, iron oxide nanoparticles [60], black phosphorus [61], PLGA nanoparticles, and layered double hydroxides (LDHs) [62,63]. Owing to their intrinsic physicochemical properties, these materials can function both as drug-delivery platforms and as therapeutically active components. For example, two-dimensional black phosphorus nanosheets have been used in sensor design, and composites comprising gold nanoparticle-modified black phosphorus and LDHs exhibit high conductivity, strong electrocatalytic activity, and a large specific surface area. LDHs have been investigated as drug carriers, including zinc–aluminum LDH for delivery of protocatechuic acid in hepatocellular carcinoma models, magnesium–iron LDH nanohybrids for adenosine delivery, and ocular delivery systems designed to prolong corneal residence. When therapeutic nanocarriers are developed, the impact of their physicochemical properties on biological behavior must be carefully considered, because particle surface characteristics and composition determine their suitability for functions such as drug loading and reactive oxygen species (ROS) scavenging [64,65,66].

#### 4.1.3. Membrane–Core Fusion

The defining step in CMN fabrication is the deposition of cell membrane fragments onto the nanoparticle core to generate a biomimetic composite structure [48,49]. Although membrane deposition is often described as a coating process, membrane–core fusion is a dynamic physicochemical event rather than a simple mechanical wrapping procedure. During coating formation, membrane-derived vesicles initially adsorb onto nanoparticle surfaces through interfacial interactions, followed by membrane deformation, vesicle rupture, membrane spreading, and fusion of membrane patches into a continuous coating layer. These sequential events determine the final architecture, integrity, and stability of the membrane coating and are influenced by the compatibility between membrane properties and nanocore characteristics [67]. Conventional coating methods include extrusion, sonication, or a combination of both. Extrusion applies mechanical forces to facilitate interactions between membrane vesicles and nanoparticle cores, whereas sonication promotes membrane integration through acoustic energy [48]. Although these methods are widely used for batch production, process parameters such as treatment duration and intensity must be tailored to the specific membrane–core pairing, often resulting in limited consistency. Repeated processing may also impair membrane function [68,69]. Importantly, complete and homogeneous membrane coverage should not be assumed for all CMN systems. Recent evidence indicates that membrane-coated nanoparticles may exist as heterogeneous populations containing fully coated, partially coated, or incompletely wrapped structures. Such variations in coating integrity may expose portions of the synthetic core and consequently influence nanoparticle stability, cellular internalization mechanisms, and biological performance. Therefore, membrane coverage efficiency and structural integrity should be considered essential quality attributes during CMN development [70]. To address these limitations, alternative strategies such as electroporation have been introduced. In particular, microfluidic electroporation can rapidly and efficiently promote membrane coating while minimizing damage to membrane functionality and is especially useful for nanoparticle cores that are difficult to coat by conventional methods [45,71]. In addition to fabrication parameters, the physicochemical properties of nanoparticle cores may also regulate membrane coating outcomes. Surface characteristics, including surface chemistry, charge distribution, curvature, and nanoscale roughness, may influence vesicle adsorption, deformation, rupture behavior, and subsequent membrane spreading. Although current evidence is mainly derived from supported lipid bilayer models rather than cell membrane-coated nanoparticles, these findings provide important mechanistic insights suggesting that nanocore surface properties should be considered when optimizing membrane coating uniformity and stability [72]. Functionalized coating strategies can also involve membrane pretreatment, including chemical modification to increase multifunctionality after coating or fusion of membranes from different cell types, such as erythrocyte and cancer-cell membranes [73,74], to generate hybrid coatings with additional biological functions [75,76]. Overall, successful membrane–core fusion requires balancing the physicochemical advantages of the nanocore with preservation of the biological functions of the membrane [46]. The membrane coating may confer source-dependent biocompatibility, immune-evasive properties, and biological recognition, whereas the synthetic nanocore provides cargo-loading capacity and tunable physicochemical properties, enabling controlled release, imaging, and therapeutic functions [77]. For example, electrostatic attraction between a positively charged core and a negatively charged hybrid membrane can stabilize the coated structure [78].

### 4.2. Functions and Advantages of Cell Membrane-Coated Nanoparticles

**Figure 3:**
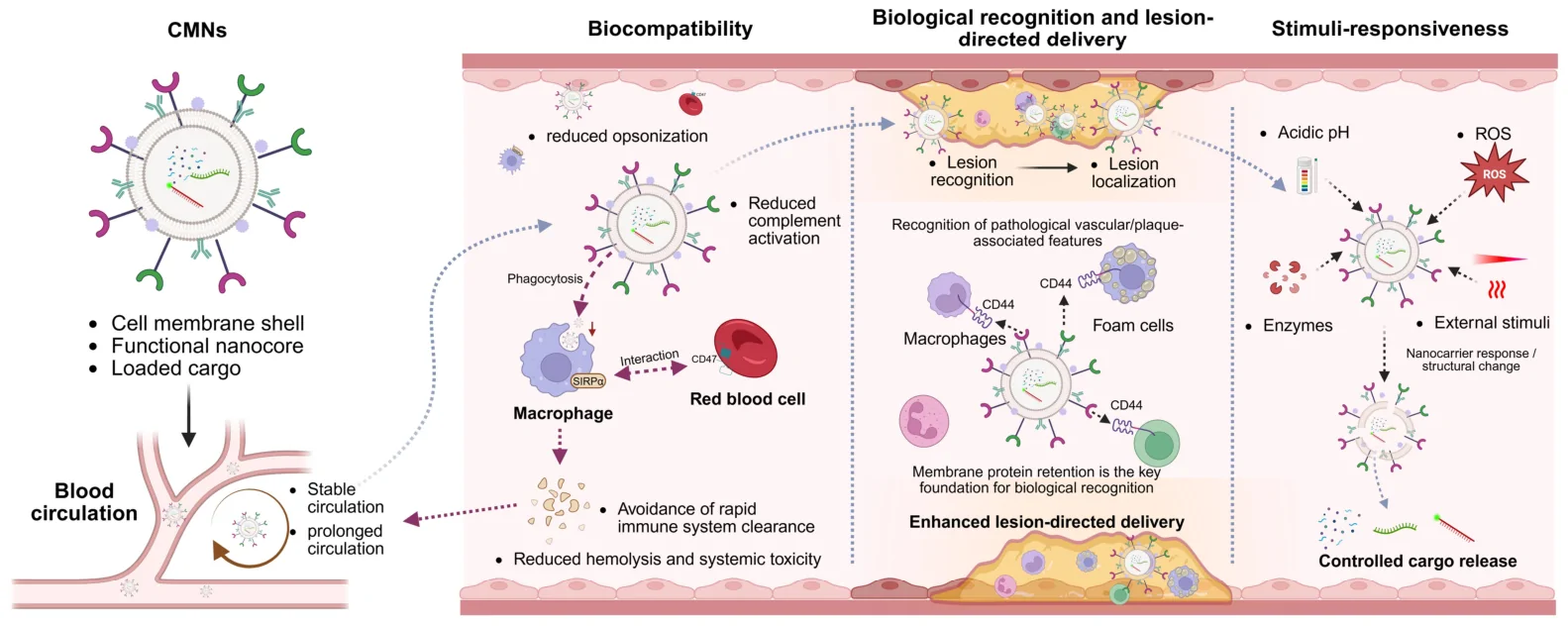
Principal functional advantages of cell membrane-coated nanoparticles (CMNs). Cell membrane coating integrates the biological properties of natural membranes with the functional versatility of engineered nanocores, providing enhanced biocompatibility, biological recognition, lesion-directed delivery, and stimuli-responsive therapeutic activation. The retained membrane components contribute to immune evasion and pathological microenvironment recognition, whereas engineered nanocores enable cargo loading, controlled release, and functional responses to disease-associated stimuli. Abbreviations: CMNs, cell membrane-coated nanoparticles; ROS, reactive oxygen species.

By incorporating the intrinsic properties of natural cell membranes, CMNs acquire multiple functional advantages, including improved biocompatibility, enhanced targeting, and stimulus-responsive behavior. These functions arise from native membrane attributes and substantially improve the performance of nanomaterials in biomedical applications (Figure 3).

#### 4.2.1. Biocompatibility

By mimicking the surface architecture of endogenous cells, membrane coating markedly improves nanomaterial biocompatibility, reduces immune activation, and prolongs systemic circulation [20,79]. The coating establishes a biomimetic interface that helps nanomaterials evade immune recognition and clearance, thereby reducing immunogenicity and systemic toxicity [80]. For example, membrane-coated nanoparticles can limit macrophage uptake and opsonization, extending their residence in the bloodstream. This effect is attributable to native membrane constituents, including phospholipids and proteins [45], which reduce immune activation against exogenous materials and improve in vivo safety [81]. Membrane coating can also increase overall particle stability. Several studies have reported prolonged circulation times and a reduced risk of hemolysis [49,82], supporting the suitability of these materials for therapeutic use.

Mechanistically, CMNs inherit self-recognition signals from donor cells, enabling them to evade immune clearance and attenuate inflammatory responses. For example, erythrocyte membrane-derived systems exhibit favorable hemocompatibility and can avoid complement-mediated elimination [83]. At the same time, the coating preserves the physicochemical properties of the nanoparticle core while adding a bioactive outer layer, thereby reducing toxicity and enhancing in vivo compatibility [48].

#### 4.2.2. Targeting Capacity

The targeting capacity of CMNs, when demonstrated by specific biological recognition, is primarily governed by two complementary mechanisms: intrinsic biological recognition mediated by retained donor-cell membrane components and ligand-mediated active targeting introduced through membrane engineering [84,85,86,87,88]. Native membrane-mediated targeting is highly source dependent. Leukocyte-, monocyte-, and macrophage-derived membranes can partially reproduce inflammation-associated adhesion and chemokine-guided recruitment to activated endothelium [28,84]. Platelet membranes retain a multivalent adhesion repertoire, including glycoprotein Ib alpha (GPIbα), glycoprotein VI (GPVI), integrin αIIbβ3, and P-selectin, enabling interactions with von Willebrand factor, exposed collagen, fibrinogen or fibrin, and inflammatory leukocytes [28,85]. In contrast, native erythrocyte membranes predominantly confer immune evasion and prolonged circulation through self-recognition and complement-regulatory molecules, but generally provide limited intrinsic plaque-specific recognition [86].

Importantly, claims of receptor-specific targeting should ideally be supported by direct mechanistic validation, such as receptor-blocking, competition assays, or other loss-of-function approaches. Increased lesion fluorescence or nanoparticle accumulation alone should not be interpreted as definitive evidence of receptor-mediated targeting. In addition, identification of membrane-associated receptors alone does not necessarily confirm the preservation of their native topology and functional competence after membrane isolation and nanoparticle coating [70]. The orientation and accessibility of membrane proteins may be influenced by membrane extraction, vesicle formation, membrane–core fusion, and nanoscale curvature effects. Importantly, preservation of a right-side-out membrane orientation is considered essential for maintaining the biological functions inherited from donor cells [89]. Therefore, functional evaluation of CMNs should extend beyond protein identification and include approaches capable of assessing receptor exposure and activity. Potential strategies include antibody accessibility assays using extracellular-domain-specific antibodies, protease protection assays, immunogold electron microscopy, receptor–ligand binding analysis, and functional blocking or competition experiments. These approaches have been widely used to evaluate membrane protein topology and extracellular accessibility in biological membrane systems [90]. For example, CD47-mediated immune evasion, VLA-4/VCAM-1-associated adhesion, GPVI/collagen recognition, and LFA-1/ICAM-1 interactions should ideally be validated through corresponding functional assays rather than inferred solely from protein retention. Such evaluations are essential for establishing a causal relationship between membrane composition and biological targeting performance.

Beyond molecular recognition and receptor functionality, vascular targeting by CMNs should also be evaluated under physiologically relevant mechanical conditions. Many platelet- and leukocyte-derived adhesion mechanisms, including GPIbα/von Willebrand factor, GPVI/collagen, and integrin-mediated interactions such as LFA-1/ICAM-1 and VLA-4/VCAM-1, are strongly influenced by vascular shear stress [91,92]. Therefore, static binding assays or cellular uptake studies performed under quiescent conditions may overestimate targeting efficiency and cannot fully predict vascular adhesion or retention during circulation [93]. Although several studies have demonstrated enhanced lesion accumulation of membrane-coated nanoparticles, only a small subset of studies have directly investigated their adhesion, rolling behavior, or retention under physiologically relevant flow conditions [94]. Future investigations should incorporate parallel-plate flow chambers, microfluidic vascular models, endothelialized vessel-on-chip systems, or ex vivo perfusion platforms to quantify rolling velocity, adhesion strength, detachment resistance, and retention under arterial shear stress [95]. Integrating biomechanical validation with molecular characterization will provide a more rigorous framework for assessing the true vascular targeting capacity of CMNs. In this context, quantitative characterization of membrane protein density, orientation, and flow-dependent adhesion performance should be incorporated into future critical quality attribute assessments for CMNs [95,96].

Active targeting can be further enhanced by displaying exogenous binding moieties on the membrane surface through covalent conjugation, lipid-anchor insertion, noncovalent assembly, metabolic labeling, or genetic engineering [87,88]. These approaches allow antibodies, targeting peptides, receptor ligands, or modular binding proteins to be incorporated while largely retaining the underlying biomimetic interface. Representative PubMed-indexed applications in atherosclerosis include a VCAM-1-targeting peptide-functionalized erythrocyte nanoplatform and erythrocyte–platelet hybrid nanoparticles modified with an antibody against C-X-C chemokine receptor 2 (CXCR2) [96,97]. Receptor-engineered hybrid membrane nanovesicles have also been used to deliver functional MER proto-oncogene tyrosine kinase (MerTK). However, this strategy is more appropriately regarded as membrane-enabled therapeutic protein delivery than as classical ligand-mediated active targeting [98]. Regardless of the modification method, ligand density, ligand orientation, membrane retention, and stability must be carefully optimized, because excessive or poorly controlled functionalization may sterically mask native membrane proteins, disrupt membrane organization, or compromise batch-to-batch reproducibility [87,88].

Stimulus-responsive designs provide an additional but mechanistically distinct layer of spatial control. Rather than directly initiating receptor-dependent lesion recognition, pathological cues such as elevated ROS, acidic pH, or disease-associated enzymes generally trigger ligand exposure, structural transformation/disassembly, or cargo release after the carrier encounters the diseased microenvironment [28,96,99,100]. Stimulus responsiveness should therefore be regarded primarily as a post-localization mechanism that improves the spatiotemporal precision of therapeutic activation, rather than as active targeting per se [11,101]. These mechanisms are discussed further in the next Section.

#### 4.2.3. Stimulus Responsiveness

Cell membrane-coated nanoparticles can be further integrated with stimulus-responsive designs that react to disease-associated microenvironmental features, such as acidic pH, elevated ROS, or specific enzymes [102,103,104]. This responsiveness arises from interactions between engineered material components and the local microenvironment. For example, thermoresponsive membrane-coated systems can increase glucose consumption and oxygen generation at defined temperatures and enable controlled drug release [105], including in response to external stimuli such as near-infrared irradiation [106]. Such systems dynamically adjust release behavior at diseased sites, improving efficacy while reducing adverse effects. pH-sensitive polymers can be combined with membrane coating to trigger drug release in acidic tumor microenvironments [107], whereas redox-responsive nanomaterials can exploit elevated glutathione concentrations to achieve selective release [108,109]. These multimodal responsive properties improve spatiotemporal control, reduce premature burst release [110], and thereby optimize therapeutic efficacy and safety [111].

## 5. Applications and Mechanistic Comparison of Biomimetic Nanosystems Derived from Different Cell Membranes in Targeted Atherosclerosis Therapy

Cell membrane-coated nanoparticles derived from different cellular sources are not functionally interchangeable. Their biological behavior depends on the membrane-associated molecules retained after fabrication and on their correspondence with specific cellular, molecular, and extracellular niches within atherosclerotic plaques [20,112]. Accordingly, membrane-source selection should be guided by the pathological niche and therapeutic objective rather than by the general concept of membrane camouflage alone.

The major membrane sources investigated for AS nanotherapy include platelets, macrophages, neutrophils, monocytes, erythrocytes, and hybrid membranes. Platelet membranes are primarily associated with vascular adhesion and thrombo-inflammatory interfaces; macrophage and monocyte membranes reproduce inflammation-associated recruitment; neutrophil membrane-coated systems retain inflammation-associated adhesion machinery that facilitates interactions with activated endothelium and supports plaque-directed cargo delivery; erythrocyte membranes predominantly provide prolonged circulation and reduced phagocytic clearance; and hybrid membranes combine complementary functions from multiple sources. T-cell-related approaches remain exploratory and are currently better regarded as targeted immunomodulatory strategies than as established T-cell membrane-coated platforms. The following sections compare these systems according to their plaque-niche correspondence, targeting mechanisms, therapeutic functions, evidence maturity, and translational limitations.

Before examining each membrane source in detail, several common barriers to clinical translation should be considered. Three overarching obstacles must be recognized. First, batch consistency and membrane heterogeneity remain major concerns. The activation and polarization state of donor macrophages—including conventionally defined unpolarized (M0), classically activated (M1-like), and alternatively activated (M2-like) states—can alter the composition and biological activity of the harvested membranes. Direct comparisons of macrophage membrane-coated nanoparticles prepared from different polarization states have demonstrated distinct cytokine-binding and anti-inflammatory properties, indicating that donor-cell state must be tightly controlled [113]. Such source-dependent heterogeneity, together with variability in membrane-coating completeness, complicates the definition of critical quality attributes and reproducible manufacturing. Quantitative studies have shown that conventional coating procedures frequently generate partially coated nanoparticles and that coating completeness materially affects targeting performance, underscoring the need for standardized manufacturing and quality-control criteria [67]. Second, scalable manufacturing and standardized quality-control criteria are lacking. Parameters used for membrane coating by extrusion or sonication, including time, intensity, and temperature, depend heavily on empirical membrane–core pairing. As a result, reproducibility is limited, costs are high, and compliance with Good Manufacturing Practice (GMP) requirements remains challenging [12,114]. Third, long-term biosafety data and clinically relevant disease models are insufficient. Most studies have relied on short-term experiments in small animals, particularly apolipoprotein E-deficient (ApoE−/−) mice [115], with limited validation in large-animal models and little evidence regarding long-term safety [116]. In particular, the risks of allogeneic immunogenicity and viral transmission associated with cell-derived membranes have not been adequately evaluated [117].

**Figure 4:**
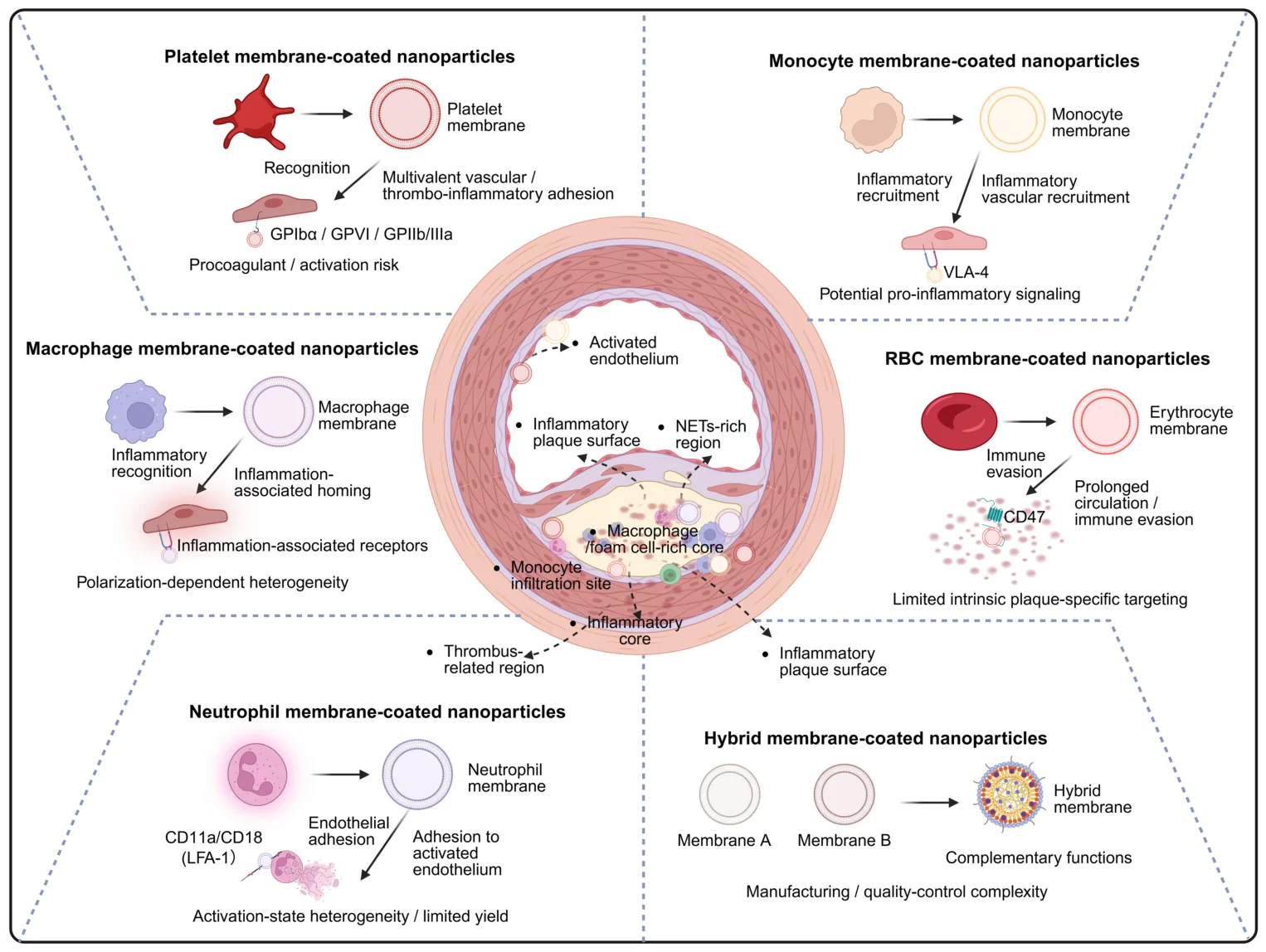
Cell membrane sources and their pathological relevance in atherosclerosis-targeted biomimetic nanomedicine. Different cell membrane sources provide distinct biological properties for lesion-directed delivery according to their native functions and associated molecular recognition mechanisms. Platelet membranes contribute thrombo-inflammatory recognition and vascular adhesion; monocyte and macrophage membranes provide inflammation-associated interactions with plaque microenvironments; neutrophil membranes facilitate adhesion to activated endothelium but are influenced by activation-state heterogeneity and limited membrane yield; erythrocyte membranes mainly enhance immune evasion and circulation persistence; hybrid membranes integrate complementary functions but introduce additional manufacturing and quality-control challenges. Appropriate membrane selection should therefore be guided by pathology-matched biological functions rather than increasing structural complexity alone. Abbreviations: GPVI, glycoprotein VI; GPIbα, glycoprotein Ibα; VLA-4, very late antigen-4; LFA-1, lymphocyte function-associated antigen-1; CD47, cluster of differentiation 47.

The following discussion evaluates each membrane system in the context of these shared translational barriers (Figure 4).

### 5.1. Platelet Membrane-Coated Nanomaterials

Platelets play a central role in the initiation and progression of AS by interacting with activated endothelial cells, recruiting inflammatory cells, and participating in plaque rupture and thrombosis. Platelet membranes retain multiple adhesion-associated proteins, including GPIbα, GPVI, integrin αIIbβ3, and P-selectin, together with immune-regulatory markers such as CD47 [91,118]. GPIbα, GPVI, and αIIbβ3 can mediate interactions with von Willebrand factor, exposed subendothelial collagen, and fibrinogen or fibrin, respectively, whereas P-selectin contributes to platelet–leukocyte interactions [91,92,93]. CD47 may primarily contribute to reduced phagocytic clearance rather than direct plaque adhesion [118]. Collectively, these multivalent interactions may support the recognition of activated endothelium, inflammatory-cell-rich regions, exposed extracellular matrix, and thrombotic components within atherosclerotic lesions [94]. The platelet membrane-coated nanoparticles (PNPs) developed by Wei et al. represent an early application of this mechanism [94]. PNPs localized not only to established plaques but also to subclinical arterial regions susceptible to plaque formation. Their fluorescence intensity was nearly tenfold higher than that of the control group (*p* < 0.0001), and positive-contrast magnetic resonance imaging (MRI) was achieved within 1 h after injection. This study demonstrated that platelet-membrane functionalization could support multicomponent lesion recognition and MRI-based detection of early atheroprone vascular regions. However, this platform primarily addressed lesion visualization and did not yet provide a therapeutic function.

Subsequent studies incorporated therapeutic modules into platelet membrane-based platforms. Zhang et al., for example, developed a platelet membrane-coated black phosphorus nanoplatform (PBP@siR@PM) that scavenged excessive ROS under oxidative conditions and delivered small interfering RNA to silence Ca^2+^/calmodulin-dependent protein kinase II gamma (CaMKIIγ). The platform enhanced macrophage efferocytosis, reduced the lipid-deposition area to 8.12%, and increased expression of the pro-efferocytic receptor MerTK by approximately 80% [119]. The conceptual strength of this strategy lies in integrating platelet membrane-mediated lesion recognition with the antioxidant properties of black phosphorus and a gene-silencing therapeutic module, thereby establishing a sequential “recognize–clear–repair” treatment paradigm. More recent studies have extended this concept. Zhou et al. developed self-luminescent platelet membrane nanoparticles (PM@CBNP) in which high plaque H_2_O_2_ concentrations triggered chemiluminescence for photodynamic therapy without an external light source, overcoming the limited tissue penetration of conventional photodynamic therapy for deeply located atherosclerotic plaques [120]. Tang et al. constructed a platelet membrane–mitochondria dual-targeting system (PM@EPPT) that increased empagliflozin delivery efficiency by 43%, expanding the potential use of hydrophobic cardiovascular drugs in targeted nanomedicine [121]. Zhu et al. used platelet membrane-coated mesoporous silica nanoparticles to co-deliver a gadolinium contrast agent and curcumin, thereby integrating MRI with anti-atherosclerotic therapy [122].

The principal advantage of platelet membrane-coated systems lies in their multivalent adhesion repertoire, which may enable simultaneous recognition of several cellular and extracellular components associated with developing and established vascular lesions. This multivalent interface may offer broader target coverage than single-ligand modification, although direct head-to-head comparisons with optimized synthetic targeting systems remain limited. Nevertheless, two major translational barriers remain. First, platelet membrane availability is limited. Platelets are readily activated and degranulated ex vivo, which can compromise membrane-protein integrity, and the cost and ethical constraints associated with large-scale procurement of human platelet membranes remain unresolved. Second, platelet membranes possess intrinsic procoagulant activity. The risk of thrombosis following systemic administration must therefore be rigorously evaluated in long-term large-animal studies.

### 5.2. Leukocyte Membrane-Coated Nanomaterials

#### 5.2.1. Macrophage Membranes

Macrophages are among the most abundant immune cells in atherosclerotic plaques. They internalize oxLDL, form foam cells, and amplify local inflammation. Their membrane proteins confer natural inflammatory homing. For example, integrin α4β1 (VLA-4) binds VCAM-1, highly expressed on inflamed endothelium, whereas the chemokine receptors C-C chemokine receptor type 2 (CCR2) and C-X3-C motif chemokine receptor 1 (CX3CR1) respond to C-C motif chemokine ligand 2 (CCL2) and C-X3-C motif chemokine ligand 1 (CX3CL1), respectively. This inflammation-associated recognition capacity is the defining advantage of macrophage membranes over many other membrane sources. Consequently, macrophage membrane-coated nanoparticles have been widely investigated for targeted diagnosis and treatment of AS. Fe_3_O_4_@M2 nanoparticles, for example, use M2 macrophage membranes to integrate dual-modal MRI/near-infrared imaging with anti-inflammatory therapy [123].

A notable recent trend is the introduction of engineered membrane strategies. Examples include genetic upregulation of CD47 to generate modified macrophage membrane (MMM)-coated nanoparticles with improved immune evasion and targeting, and integrin α9β1-modified membrane-coated carriers designed as molecular imaging probes for precise recognition of vulnerable plaques [89,124]. At the therapeutic level, the retinoic acid-loaded macrophage membrane-biomimetic liposome (R@MLP) developed by Cai et al. is representative. Native SIRPα on the membrane blocks the CD47–SIRPα axis to enhance efferocytosis, whereas retinoic acid upregulates ATP-binding cassette transporter A1 (ABCA1) and ATP-binding cassette transporter G1 (ABCG1) to promote cholesterol efflux. These two mechanisms jointly improve the plaque inflammatory microenvironment by enhancing both efferocytosis and cholesterol export [125]. Dong et al. fused macrophage and erythrocyte membranes and added hyaluronic acid functionalization. This platform simultaneously inhibited nuclear factor kappa B (NF-κB)-associated lipid influx and activated AMP-activated protein kinase (AMPK)/mechanistic target of rapamycin (mTOR)-mediated cholesterol efflux, enabling sequential early suppression and late-stage regression of disease [126].

The distinctive advantage of macrophage membrane systems is their ability to reproduce macrophage function in situ. By disguising nanoparticles as macrophages, the membrane enables targeted homing while allowing active immunomodulation through proteins such as SIRPα. However, membrane heterogeneity is a major limitation. Protein-expression profiles differ substantially among M0, M1, and M2 macrophage membranes; therefore, targeting efficiency and immunomodulatory effects depend strongly on the selected subtype, a variable that remains insufficiently controlled in most studies. Clinical translation will require membrane-source quality-control standards based on flow cytometry or proteomics to ensure consistent receptor expression across batches.

#### 5.2.2. Neutrophil Membranes

Neutrophil membrane-coated nanoparticles (NM-NPs) transfer selected inflammation-associated surface properties of neutrophils to synthetic nanocarriers. Unlike living neutrophils, however, NM-NPs do not actively migrate along chemokine gradients. Their lesion-directed accumulation is primarily attributed to retained leukocyte adhesion machinery, particularly cluster of differentiation 18 (CD18)-containing β2 integrins, including leukocyte function-associated antigen-1 (LFA-1), which interact with intercellular adhesion molecule-1 (ICAM-1) upregulated on activated endothelial cells. This biomimetic interface can simultaneously enhance adhesion to inflamed vascular regions, reduce phagocytic clearance, and improve local delivery of therapeutic cargos [127,128,129,130]. Liu et al. [127] provided direct mechanistic evidence by constructing a neutrophil membrane-coated zeolitic imidazolate framework-8 (ZIF-8) platform, AM@ZIF@NM, for delivery of an antisense oligonucleotide against microRNA-155 (miR-155). The membrane coating enhanced recognition of plaque endothelial cells through the CD18–ICAM-1 interaction, whereas the ZIF-8 core promoted oligonucleotide loading and endolysosomal escape. Subsequent miR-155 silencing restored B-cell lymphoma 6 (BCL6) expression and suppressed RELA/NF-κB-associated CCL2 and ICAM-1 expression, thereby attenuating vascular inflammation and atherosclerotic lesion development [127].

Beyond nucleic-acid delivery, neutrophil membrane coating has been integrated with intracellular protein delivery and autophagy regulation. Liang et al. [128] screened an autophagy-inducing polypyridinium, P5c, capable of delivering superoxide dismutase (SOD) and catalase (CAT) into the cytosol of macrophages. The resulting P5c/SOD/CAT nanoparticles were further coated with neutrophil membranes to generate NeuM@P5c/S/C. This platform reduced intracellular ROS, promoted macrophage autophagy, inhibited foam-cell formation, and favored anti-inflammatory macrophage polarization. In ApoE−/− mice, treatment reduced senescent-cell infiltration within plaques and improved systemic immune-organ architecture, illustrating how neutrophil membrane-mediated delivery can be combined with protein replacement and intracellular pathway regulation rather than serving merely as a surface camouflage [128]. The study therefore extends the functional scope of NM-NPs from conventional drug delivery to simultaneous correction of oxidative stress, defective autophagy, and macrophage dysfunction.

The modularity of this membrane interface has also been demonstrated with small-molecule and catalytic nanotherapeutics. Zhang et al. [129] developed the simvastatin-loaded neutrophil membrane-camouflaged nanoplatform NNPST, which exhibited prolonged circulation, enhanced plaque accumulation, and fluorescence/magnetic resonance dual-modal lesion visualization. In ApoE−/− mice, NNPST reduced aortic plaque burden primarily by suppressing local inflammatory macrophage responses [129]. More recently, Wu et al. [130] constructed a neutrophil membrane-coated confined cascade nanoreactor composed of ultrasmall Prussian blue nanoparticles (USPBs) and selenium-doped dendritic mesoporous silica nanoparticles (SeDMSNs), termed USPB@SeDMSN@NM. USPBs supplied SOD- and CAT-like activities, whereas selenium-doped dendritic mesoporous silica provided glutathione peroxidase-like activity. The neutrophil membrane retained integrin β2 and LFA-1 and enhanced interactions with ICAM-1-expressing activated endothelial cells and inflammatory macrophages. The resulting system prolonged circulation, increased plaque accumulation, scavenged multiple ROS, reduced inflammatory cytokine production and lipid accumulation, promoted M1-to-M2 macrophage polarization, and attenuated oxidative DNA damage and p53/p21-associated cellular senescence [130]. Collectively, these studies show that neutrophil membrane coating is compatible with oligonucleotide delivery, intracellular enzyme delivery, conventional small-molecule therapy, and multienzyme-like nanocatalysis.

The neutrophil membrane should not necessarily be regarded as a biologically inert targeting shell. In a non-atherosclerotic inflammatory model, payload-free NM-NPs were shown to sequester inflammatory cytokines and chemokines, interfere with leukocyte adhesion to activated endothelium, and influence macrophage phenotype after phagocytic uptake, partly through membrane-associated phosphatidylserine [131]. These observations suggest that membrane-derived anti-inflammatory activity may cooperate with the therapeutic core; however, this effect has not been systematically established in atherosclerosis and should not be assumed without appropriate controls. Future studies should therefore include payload-free NM-coated nanoparticles and composition-matched synthetic membrane controls to distinguish the respective contributions of the membrane, nanocore, and cargo. Translation will also require control of neutrophil activation state, adhesion-molecule abundance, residual intracellular or granule-associated components, coating completeness, and batch-to-batch consistency.

#### 5.2.3. Monocyte Membranes

Monocyte membrane-coated nanomaterials exploit the homing behavior of monocytes to target inflamed vascular sites [132]. The Monocyte Cell Membrane-Coated 1,8-Cineole Biomimetic Delivery System (MM-CIN-BDS) combines monocyte membranes with diethylaminoethyl dextran to deliver 1,8-cineole, substantially improving cellular uptake and reducing plaque burden [133]. Separately, blockade of the CD47–SIRPα antiphagocytic checkpoint has been shown to restore defective efferocytosis and attenuate atherosclerosis, although this mechanism has not yet been directly established for monocyte membrane-coated nanomaterials [134]. Monocyte membrane systems can preferentially recognize activated endothelial cells and sites of monocyte infiltration within plaques and are therefore well suited for delivering anti-inflammatory or lipid-modulating agents. However, monocytes themselves participate in plaque inflammation, and whether their membranes could introduce additional proinflammatory signals requires further investigation.

#### 5.2.4. T-Cell-Targeted Nanotherapeutic Strategies

Direct evidence for T-cell membrane-coated nanoparticles in atherosclerosis remains limited. Current studies primarily focus on nanocarriers that target specific T-cell subsets or modulate T-cell-associated immune checkpoints. Because T helper 1 (Th1) and T helper 17 (Th17) cells generally promote inflammation, whereas regulatory T cells exert protective effects, future nanotherapeutic strategies should aim to achieve subset-selective modulation rather than nonspecific T-cell inhibition [135]. Immune-checkpoint interventions also require careful evaluation, as experimental cytotoxic T-lymphocyte-associated protein 4 (CTLA-4) blockade has been shown to aggravate plaque inflammation and atherosclerosis progression in hyperlipidemic mice [136].

### 5.3. Erythrocyte Membranes

Erythrocyte membranes generally lack intrinsic lesion-specific targeting but preserve native red-blood-cell surface markers, particularly CD47, which contributes to immune evasion and prolonged systemic circulation [86]. As a result, intravenously administered nanoparticles can circulate for prolonged periods and avoid immune clearance [137]. Recent studies have expanded their functional scope. Zhong et al. reported a “plug-and-play” erythrocyte nanoplatform whose surface could be functionalized to target atherosclerotic lesions [96]. However, native erythrocyte membrane-coated nanoparticles generally lack intrinsic plaque-specific targeting [15,138]. Without additional targeting ligands or complementary membrane components, they mainly rely on prolonged circulation and passive lesion access, which may be insufficient for efficient accumulation in early or stable plaques [96,139].

### 5.4. Hybrid Membranes

Each single-source membrane has inherent limitations: erythrocyte membranes provide prolonged circulation but lack active targeting; platelet membranes exhibit strong lesion targeting but may promote coagulation; and macrophage membranes display precise inflammatory homing but substantial heterogeneity. Hybrid-membrane strategies seek to achieve functional complementarity by physically fusing or genetically integrating components from two or more cell membranes. Representative advances in the AS field are summarized below.

#### 5.4.1. Erythrocyte–Platelet Hybrid Membranes

The red blood cell–platelet hybrid membrane-coated nanoparticles ([RBC–P]NPs) developed by Dehaini et al. combined the prolonged circulation of erythrocyte membranes (half-life, 51.8 h) with the disease-targeting properties of platelet membranes [140]. Huang et al. further developed RBC–P]NPs modified with an antibody against CXCR2. By blocking the C-X-C motif chemokine ligand 8 (CXCL8)–CXCR2 axis, the system suppressed p38α-mediated M1 polarization, reduced plaque volume, and improved plaque stability while avoiding the bleeding risk associated with conventional antiplatelet drugs [97]. A 2026 study used platelet-rich plasma-derived extracellular vesicles (PEVs) to deliver the poly(ADP-ribose) polymerase (PARP) inhibitor niraparib and incorporated Ca(HCO_3_)_2_-mediated gas generation to integrate ultrasound imaging with therapy. Notably, the PEVs not only served as targeted carriers but also attenuated oxidative stress, thereby enhancing the therapeutic activity of the PARP inhibitor and providing proof of concept for a “carrier-as-therapy” paradigm [141].

#### 5.4.2. Macrophage–Genetically Engineered Hybrid Membranes

Qiu et al. [98] developed magnetically guided hybrid membrane nanovesicles (HMNVs) by fusing membranes from MerTK-overexpressing macrophages with membranes from transferrin receptor-overexpressing human embryonic kidney 293T (HEK293T) cells. With superparamagnetic iron oxide-mediated magnetic navigation, the system delivered MerTK protein to target sites, restored efferocytosis under diabetic conditions independently of metabolic regulation, and reduced the liver and spleen accumulation typical of conventional carriers [98].

#### 5.4.3. Macrophage Membrane–Liposome Hybrid Systems

Chen et al. [142] used genetic engineering to generate a hybrid membrane overexpressing VLA-4 and fused it with an ROS-responsive liposome (TK-VLA@TAT). A tetrahedral nucleic acid framework was used to deliver atorvastatin, enabling ROS-responsive and precise drug release within the plaque microenvironment [142]. Another hyaluronic acid (HA)-modified macrophage membrane–liposome hybrid nanoplatform, termed HA-ML@ES NPs, co-delivered shikonin and evolocumab to restore macrophage cholesterol-flow homeostasis and reprogram macrophage polarization while simultaneously repairing endothelial dysfunction. In homocysteine-induced ApoE−/− mice, the platform markedly reduced plaque burden and increased collagen content and fibrous-cap thickness, indicating improved plaque stability and supporting the value of coordinated multitarget intervention for the complex pathology of AS [143].

Hybrid-membrane strategies represent the most highly integrated design concept currently available in the field of membrane biomimetics. Their central logic is to modularly combine targeting functions provided by platelet or macrophage membranes, prolonged circulation provided by erythrocyte membranes, and intelligent responsiveness provided by engineered membranes or ROS-responsive modules. This approach enables multitask synergy that cannot be achieved with a single membrane source. However, design complexity creates substantial translational challenges: each additional membrane component exponentially increases the number of interacting quality-control parameters, making GMP-compliant production progressively more difficult [144]. A key future breakthrough may therefore lie in standardized modular-assembly processes rather than de novo design of each hybrid-membrane combination. Although hybrid-membrane systems can integrate complementary cellular functions, their increasing structural complexity places greater demands on coating completeness, membrane-fusion efficiency, and product consistency. Quantitative studies have shown that conventional membrane-coating procedures frequently produce partially coated nanoparticles, underscoring the need for standardized fabrication and quality-control methods before clinical translation [70,145].

### 5.5. Section Summary and Translational Outlook

The membrane systems described above differ in their targeting interfaces, delivery modes, and source-specific limitations. Neutrophil membrane-coated systems provide an inflammation-associated interface for the delivery of oligonucleotides, intracellular enzymes, small-molecule drugs, and catalytic nanozymes; however, their translational development remains constrained by membrane sourcing, donor-cell activation state, coating quality, and batch consistency. Hybrid systems such as HMNVs and TK-VLA@TAT may support mechanistically justified multitask therapy but impose more demanding manufacturing and quality-control requirements.

A major translational bottleneck is the lack of reproducible, scalable manufacturing and robust safety-assessment frameworks. Future translational efforts should therefore prioritize three areas. First, proteomics-based quality-control standards should be established to define the critical quality attributes of each membrane source. Second, scalable membrane-coating technologies such as microfluidic electroporation should be developed to enable automated and standardized production. Third, pharmacokinetics and long-term safety should be evaluated in large-animal models, including pigs and non-human primates (Table 1).

## 6. Conclusions and Perspectives

Cell membrane-coated nanoparticles have advanced atherosclerosis nanotherapy from passive drug delivery toward biologically guided lesion localization and selective recognition. The studies reviewed here show that different membrane sources are suited to distinct plaque niches rather than forming a simple hierarchy of performance. Platelet membranes are particularly effective for recognizing activated endothelium and thrombo-inflammatory interfaces; monocyte and macrophage membranes reproduce inflammatory-cell recruitment and facilitate access to foam-cell-rich regions; neutrophil membrane-coated systems provide inflammation-associated adhesion to activated endothelium and support the delivery of diverse therapeutic cargos, including nucleic acids, enzymes, small molecules, and catalytic nanomaterials; erythrocyte membranes mainly provide immune evasion and prolonged circulation; and hybrid membranes integrate complementary functions when a single source is insufficient. Accordingly, membrane selection should follow a pathology-matched, minimum-sufficient-complexity principle. Greater structural complexity should not be regarded as inherently superior unless each additional component provides a clear and non-redundant therapeutic benefit.

Despite encouraging preclinical results, the current evidence remains insufficient for direct clinical translation. Most studies are short-term proof-of-concept experiments in ApoE−/− or related small-animal models, and commonly rely on plaque area, fluorescence accumulation, or inflammatory markers as endpoints. Importantly, fluorescence-based biodistribution analysis alone cannot fully define the spatial fate of biomimetic nanomaterials within atherosclerotic lesions [146,147]. Increased fluorescence signals may reflect vascular retention, endothelial association, extracellular deposition, plaque penetration, or cellular internalization, but cannot distinguish these processes at the microscopic level [148]. Therefore, future studies should integrate hierarchical imaging strategies combining whole-body/ex vivo fluorescence imaging with high-resolution localization approaches. Confocal microscopy and immunofluorescence imaging can provide information regarding nanoparticle distribution relative to endothelial cells, macrophages, smooth muscle cells, and other plaque components, whereas intravital microscopy, two-photon imaging, super-resolution microscopy, and electron microscopy-based approaches may further resolve nanoparticle localization at cellular or subcellular scales [149]. High-resolution imaging studies, such as the work reported by Beldman et al. [147], have demonstrated the value of spatially resolved imaging approaches for dissecting nanoparticle localization within atherosclerotic plaques. Establishing standardized spatially resolved imaging workflows will therefore be critical for accurately evaluating lesion targeting, plaque penetration, and cell-specific delivery of future CMN platforms. Clinically meaningful features such as fibrous-cap integrity, necrotic-core expansion, intraplaque hemorrhage, thrombogenicity, plaque rupture, and cardiovascular events are evaluated less consistently. Moreover, many studies compare membrane-coated nanoparticles only with uncoated formulations, making it difficult to distinguish the specific contributions of the membrane, nanocore, cargo, and responsive module. To address this challenge, future studies should adopt modular and component-resolved experimental designs to establish causal relationships between individual components and therapeutic outcomes [117]. Appropriate control groups should include uncoated nanoparticles to evaluate nanocore-dependent effects, payload-free membrane-coated nanoparticles to determine membrane-derived biological contributions, free drug controls to assess the advantage of nanodelivery, and component-ablation designs in which specific functional modules are selectively removed (e.g., membrane depletion, ligand removal, or omission of responsive elements). Such systematic comparisons are particularly important for hybrid membrane systems and multifunctional nanoplatforms, where synergistic effects may arise from multiple interacting components. Establishing these standardized evaluation frameworks will be essential for determining whether increased structural complexity provides a genuine therapeutic advantage or merely introduces additional manufacturing and translational challenges [117]. Translation is further limited by variability in membrane-protein abundance, orientation, glycosylation, and biological activity after extraction and coating, as well as by donor differences, cell activation states, and biomolecular-corona formation. These issues are particularly relevant to the potential procoagulant activity of platelet membranes, macrophage-polarization heterogeneity, activation-state-dependent heterogeneity, limited membrane yield, and uncertain intrinsic bioactivity of neutrophil membranes, and the manufacturing complexity of hybrid membranes.

Future development should therefore prioritize standardized comparison, quality control, and clinically relevant validation. Head-to-head studies should include free drug, optimized synthetic carriers, single-ligand-targeted nanoparticles, uncoated cores, and payload-free membrane controls, while reporting both delivery efficiency and plaque-stabilizing outcomes. Critical quality attributes should include source-specific membrane markers, membrane orientation and accessibility, coating completeness, membrane-to-core ratio, fusion efficiency, cargo loading, release kinetics, sterility, and endotoxin burden. Human-relevant vascular models, patient-derived plaque tissues, and large-animal studies should be incorporated to evaluate pharmacokinetics, coagulation, complement activation, immunogenicity, infection susceptibility, biodistribution, and long-term toxicity. Scalable closed-system manufacturing, including continuous-flow microfluidic coating and in-line monitoring, will also be essential for GMP-compatible production. In the near term, comparatively simple and well-characterized single-source membrane platforms may offer a more realistic translational route, whereas hybrid membranes should be reserved for mechanistically justified multitask therapy. Progress in this field will ultimately depend less on adding functions than on demonstrating reproducible product quality, clinically meaningful plaque stabilization, and a clear safety and efficacy advantage over existing treatments. Addressing membrane-source and activation-state heterogeneity will require multidisciplinary strategies integrating standardized biological sourcing, advanced molecular characterization, and computational approaches [117]. For example, controlled donor-cell selection, defined polarization protocols, and multi-omics profiling may help establish source-specific critical quality attributes [67,80]. In addition, artificial intelligence and machine-learning algorithms may facilitate the integration of proteomic, transcriptomic, physicochemical, and functional datasets to predict membrane performance, optimize fabrication parameters, and improve batch-to-batch quality control [150,151]. Such approaches may accelerate the transition of CMNs from empirical biomimetic designs toward standardized and clinically translatable therapeutic platforms.

## Figures and Tables

**Table 1:** Comparative characteristics, recognition and delivery mechanisms, and translational limitations of representative membrane-based biomimetic nanoplatforms for atherosclerosis.

Membrane Source	Recognition/Delivery Mechanism	Key Membrane Proteins	Principal Advantage	Major Limitation	References
Erythrocyte membrane	Immune evasion and prolonged circulation with passive lesion access; plaque-specific targeting generally requires ligand engineering or hybridization.	CD47	Prolonged circulation and reduced phagocytic clearance.	Lack of active targeting	[78,81,125,126]
Platelet membrane	Multivalent adhesion to von Willebrand factor, exposed collagen, fibrinogen/fibrin, and inflammatory leukocytes.	GPIbα, GPVI, integrin αIIbβ3, P-selectin, CD47.	Multivalent recognition of activated endothelium, exposed extracellular matrix, and thrombo-inflammatory components; recognition of early susceptible lesions	Ex vivo activation and potential procoagulant risk	[77,103,104,105,107,108,109,110,111]
Macrophage membrane	Inflammation-associated recognition mediated by retained adhesion and chemokine-related receptors, including CCR2/CX3CR1, together with VCAM-1-associated adhesion.	VLA-4, CCR2, CX3CR1, SIRPα	Inflammation-associated lesion recognition with potential immunomodulatory functions.	Marked heterogeneity among M0/M1/M2 receptor profiles	[12,76,97,112,113,114]
Neutrophil membrane	Inflammation-associated adhesion and lesion accumulation mediated primarily by CD18-containing β2 integrins/LFA-1 interacting with ICAM-1 on activated endothelium.	CD11a/CD18 (LFA-1); other molecules are platform dependent	Plaque-directed accumulation and delivery of diverse cargos, including oligonucleotides, antioxidant enzymes, small-molecule drugs, and catalytic nanozymes	Activation-state-dependent membrane heterogeneity, limited membrane yield, uncertain contributions of intrinsic membrane bioactivity, and demanding coating-quality control	[94,116,117,118,119]
Monocyte membrane	Inflammation-associated recognition of activated endothelium mediated by adhesion molecules and chemokine-related receptors.	VLA-4, CCR2	Preferential accumulation at endothelial activation sites.	Potential introduction of additional proinflammatory signals	[120,121]
Hybrid membrane	Multimodule synergy (biological recognition + prolonged circulation + stimulus responsiveness)	Integrated proteins from multiple sources	Integration of complementary functions from multiple membrane or engineered modules	Exponentially increasing quality-control complexity and difficult GMP manufacturing	[82,83,115,127,129,130,132,133]

Abbreviations: CD47, cluster of differentiation 47; GPIbα, glycoprotein Ibα; GPVI, glycoprotein VI; VLA-4, very late antigen-4; CCR2, C-C chemokine receptor type 2; CX3CR1, C-X3-C motif chemokine receptor 1; SIRPα, signal regulatory protein alpha; LFA-1, lymphocyte function-associated antigen-1.

## Data Availability

No new data were created or analyzed in this study. Data sharing is not applicable to this article.
